# A Public Health Perspective on Screening for Psychosis Within General Practice Clinics

**DOI:** 10.3389/fpsyt.2019.01025

**Published:** 2020-01-31

**Authors:** Leda Kennedy, Kelsey A. Johnson, Joyce Cheng, Kristen A. Woodberry

**Affiliations:** ^1^Department of Psychiatry, Beth Israel Deaconess Medical Center, Boston, MA, United States; ^2^Columbia Irving Medical Center, New York State Psychiatric Institute, New York, NY, United States; ^3^Wellesley College, Wellesley, MA, United States; ^4^Center for Psychiatric Research, Maine Medical Center, Portland, ME, United States

**Keywords:** adolescents, prevention, primary care, clinical high risk, global mental health

## Abstract

Screening for major mental illness in adolescents and young adults has lagged behind screening for physical illness for a myriad of reasons. Existing pediatric behavioral health screening tools screen primarily for disorders of attention, disruptive behaviors, depression, and anxiety. A few also screen for substance use and suicide risk. Although it is now possible to reliably identify young people at imminent risk for a psychotic disorder, arguably the most severe of mental illnesses, general practitioners (GP) rarely screen for psychotic symptoms or recognize when to refer patients for a specialized risk assessment. Research suggests that barriers such as inadequate knowledge or insufficient access to mental health resources can be overcome with intensive GP education and the integration of physical and mental health services. Under the lens of two public health models outlining the conditions under which disease screening is warranted, we examine additional evidence for and against population-based screening for psychosis in adolescents and young adults. We argue that systematic screening within general health settings awaits a developmentally well-normed screening tool that includes probes for psychosis, is written at a sufficiently low reading level, and has acceptable sensitivity and, in particular, specificity for detecting psychosis and psychosis risk in both adolescents and young adults. As integrated healthcare models expand around the globe and psychosis-risk assessments and treatments improve, a stratified screening and careful risk management protocol for GP settings could facilitate timely early intervention that effectively balances the benefit/risk ratio of employing such a screening tool at the population level.

## Introduction

Adolescence and early adulthood is the period of peak incidence for major mental illnesses (1). A large body of evidence now suggests that early intervention can reduce the duration of untreated illness and improve treatment outcomes for individuals in the initial stages of a major psychotic disorder (2). Improved detection of the early signs and symptoms emerging prior to or during this period has particular potential to improve long-term outcomes.

In spite of this evidence, even intervention in the first year or two following a first episode of psychosis (FEP) has proved challenging. Many of the initial symptoms of psychosis are not identified as such during the first months and years (2, 3). This is particularly troubling because the period preceding and including the first 5 years of illness is the window in which one third of suicides are completed, violent behavior may emerge, and impairments in neurocognition and functioning begin or worsen (2). As a result, a number of countries have developed early psychosis treatment programs for help-seeking youth. Yet, the fact remains that most youth who develop major psychotic disorders suffer for years before accurate diagnosis and treatment. Non-help-seeking but symptomatic youth are particularly at risk for delays in care (4, 5). If the promise of early intervention is to be realized, detection of emerging psychosis in this initial window must improve and reach those who need help but are afraid or uncertain how to seek it.

One of the major advances of the last three decades has been the identification of recognizable syndromes prodromal to schizophrenia-spectrum disorders (6–8). Because not all who have these syndromes transition to a psychotic disorder, syndromic individuals are broadly considered at “clinical high risk” (CHR). The majority of these youth have had psychotic-like symptoms for months to years prior to syndrome identification (9), and subtle, insidious, but not overtly psychotic symptoms for even longer (3, 10–12). A number of the earliest symptoms, such as insomnia, might be expected to prompt help-seeking from general practitioners (GP), who, if they have followed their patients over years, are well positioned to note gradual functional declines that might otherwise go unnoticed. For these reasons, one might expect GP to be the early frontier to psychosis detection.

## History of Global Efforts

Involving GP in the early detection of psychosis is not a new idea. Falloon and colleagues (13) in the United Kingdom (U.K.) conducted landmark studies of GP system interventions beginning in the early 1990's. In fact, they found that not only were GP a fruitful target for identifying emerging psychosis, but that formal screening in the context of GP services integrated with family and specialized mental health resources was associated with reduced incidence of schizophrenia in targeted communities (13). In this “Buckingham Project,” GP and nurses were trained to inquire about specific and nonspecific risk factors such as insomnia, hallucinations, and grandiosity in all patients. A mental health professional was directly available to the GP office to facilitate a faster and more efficient pathway to care for positive screens. In Switzerland, Platz et al. (14), building on key components of Falloon's early work, found that intensive training focused on helping GP recognize insidious onset was associated with significantly improved knowledge and referral to specialized psychosis services. In fact, over half of referrals to this clinic contacted GP for help along their path to care, and 35% identified GP as their first point of contact. Particularly impressive, these referrals resulted largely from early help-seeking for insidious and nonspecific concerns rather than psychotic symptoms (15). In short, “sensitization” worked (16).

French and colleagues (17), in the U.K, tested a screening “checklist” designed to help GP evaluate help-seeking individuals. Unfortunately, it had poor specificity for detecting true psychosis risk, even in this population. Other U.K researchers, Perez and colleagues (18), compared the efficacy of low-intensity GP outreach (informational leaflets) against a high-intensity training and education campaign. Consistent with the model used in Buckingham, the intensive campaign that emphasized a more integrated relationship between physical and mental healthcare yielded more referrals and was a more clinically and cost-effective referral paradigm than traditional care (19). The relevance of GP practices to early intervention in psychosis has been indirectly exemplified by other literature. GP referral rates to specialized psychosis services were low in a Swiss study in which the training of GP and integration of physical and mental health services were absent (20). By contrast, a Canadian program using extensive community outreach found that 36% of help-seeking contacts prior to a FEP were with a GP (21). Furthermore, an impressive review of nearly 100,000 records of primary care visits in the UK confirmed the predictive value of non-specific concerns (suicidal ideation, obsessive-compulsive symptoms, and social isolation) with the development of a psychotic disorder within the subsequent 5 years, and identified a rise in medical visits for such complaints in the 3 months prior to a psychosis diagnosis (3). In a study of three regions of Norway, Bratlien and colleagues (4) found that self-reported eating disorder issues at ages 15 and 16, but not rates of health service use, were associated with higher rates of subsequent psychosis treatment. The potential role for GP in recognizing early and non-specific risk factors is clear, even if their role in the pathway to specialized services may vary across international boundaries (21, 22).

## A Public Health Perspective on Screening for Psychosis Among General Practitioners

In spite of the pioneering work noted above, delays to accurate diagnosis and treatment continue, particularly for earlier and insidious onsets (20). The potential for early detection within primary healthcare systems remains unrealized. For GP, limited knowledge and skills in recognizing the early signs of mental illness may be a critical barrier to early intervention. This barrier may be overcome by a key element of the Buckingham project: universal screening. The World Health Organization (WHO) has clear guidelines on where and when to implement screening, a number of which are clearly fulfilled for schizophrenia-spectrum disorders (**Table 1**; 23). The remaining WHO criteria pose important and serious challenges, which if taken on, will spawn necessary growth in the early intervention effort. Critical steps must be taken before screening for psychosis can be wisely implemented.

**Table 1:** World Health Organization Guidelines, Abbreviated (22).

WHO guidelines for disease screening tools
1. Condition must be an important health problem.
2. An accepted treatment should be available.
3. Facilities must be available for diagnosis and treatment.
4. There should be a stage of recognizable early symptoms.
5. There should be a suitable test or examination.
6. The test should be acceptable to the population.
7. The natural history of the condition, including development from prodromal to declared disease, should be adequately understood.
8. There should be a policy on whom to treat.
9. The cost of case finding (including diagnosis) should be economically balanced in relation to possible overall costs of medical care.
10. Case finding should be ongoing and not just a single time effort.

The first four WHO criteria are easily met for schizophrenia and other psychotic-spectrum disorders. These disorders have an unquestionable impact on both individual and public health [criterion 1; (24, 25)]. There are well-established and generally acceptable, albeit imperfect, treatments available [criterion 2; (26–28)]. Similarly, most countries have established mental health systems and facilities for treating serious mental illness, even if access and quality may be inadequate [criterion 3; (29)]. GPs have different roles in early treatment-seeking and referrals to specialized care depending on individual health policies and systems (22, 30). Given proper training and connections to mental health resources, GPs may be some countries' main line of defense in spotting early psychosis (3, 21). The last 30 years have seen a major step forward in clarifying the early syndromes that precede psychotic disorders. Both retrospective and prospective studies have identified symptoms and biological markers characteristic of this prodromal stage and predictive of disease onset; risk calculators are continually being improved [criterion 4; (31, 32)]. Thus, we believe criterion 4 has been met, particularly for schizophrenia-spectrum disorders.

Criteria 5 and 6 call for a suitable test that is acceptable to the population in which it is performed. There are certainly established diagnostic criteria and structured interviews to diagnose psychotic-spectrum disorders [e.g., Structured Clinical Interview of Diagnostic and Statistical Manual of Mental Disorders, 5th Edition (DSM-5), The Structured Clinical Interview for DSM-5 (SCID-5); (33)]. In addition, structured interviews are available to reliably identify youth with a 35% risk of imminent transition to a psychotic disorder (34). None of these are suitable and acceptable for use with a general population sample. They require substantial training and administration time. Self-report is likely to be the only cost-effective way to screen at the population level [(35); criterion 9)]. Several self-report screening tools have been developed, some with fairly good psychometric qualities (36, 37). However, most have been untested in general population, particularly adolescent samples, or have unacceptable rates of false positives relative to interview validation. Furthermore, in spite of data showing that age is a key factor in the frequency of psychotic-like experiences [e.g., (38)], there are almost no age-specific norms or thresholds for these screens. Self-report tools that have been tested in adolescent samples [e.g., the CAPE; (39)], are not written at an appropriate reading level for a general population sample of adolescents, despite the fact that this is the age range in which the incidence of psychotic disorders peaks (40). This is a substantial barrier as querying complex and abstract self-observations is inherently difficult to accomplish with simple language and short sentences. Efforts to prospectively probe early basic symptoms and self-disturbances have illustrated this challenge (41), yet refined questions continue to be tested (42). On a more encouraging note, the natural course and history of psychotic spectrum disorders is becoming ever clearer, in spite of the limited progress on specific causal mechanisms [criterion 7; (43)].

To satisfy criterion 8, there must be a policy on whom to treat. There is broad international consensus on the treatment of psychotic disorders, particularly within the first years of symptom onset (2). Although consensus on the treatment of CHR youth is still lacking largely due to clinical heterogeneity and challenges addressing early functional deficits (44), published guidelines do exist supporting specialized treatment in this stage of illness (32). Finally, criterion 10 indicates that screening must be ongoing. This remains an aspirational goal in the early detection of psychosis. Yet, if progress continues with screening tools and mental health service reform, it is not unrealistic to expect that youth, particularly those with known risk factors or changes in behavior or functioning, be screened on a repeated basis throughout the period of peak risk.

Aside from the WHO criteria, there is another model used to assess the appropriateness of screening called “The Balance Approach.” This model suggests that the benefits of early detection should outweigh the risks of screening (45). It implores researchers to be conscientious of over-diagnosis, and to avoid measures that yield too many false positives. Prominent voices in the field of early intervention have argued against screening for psychosis at a population level, due primarily to concerns that transient or benign symptoms would be overpathologized [e.g., (46, 47)]. To address this important concern, any response to positive screens must begin with a general mental health-focused inquiry. In support of this approach is the fact that “false positive” psychosis screens are often “true positive” mental health screens. Perez et al.'s (18) research found that 68% of these individuals had other mental health conditions which required treatment. Systematic attention to balancing the risk of delayed identification with the risk of over-pathologizing needs to be central to any public screening effort. A stratified approach, ranging from a general mental health assessment to the skilled inquiry into the content, meaning-making, and distress associated with reported psychotic-like experiences, has potential to achieve this balance and protect low risk youth. Ideally, psychosis screening items would be embedded in general mental health screens.

## Next Steps

With the increasing integration of physical and mental health care and the growing evidence for early intervention, it is time to overcome the remaining barriers to psychosis screening in adolescents and young adults. Major mental illnesses are an important health problem, for which too much of the care is provided in the chronic phases. Careful stratification of both risk and response could minimize harm to the majority of individuals at relatively low risk while maximizing the benefits to those at higher risk or with diagnosable psychotic disorders. GP clinics with integrated mental health services are ideal settings for ongoing screening and referral of these patients.

Toward this end, we identify the following steps:

1. A concerted effort is needed to improve and test self-report screening items for adolescents and young adults. We must collect normative data on the range and frequency of psychotic-spectrum experiences, including unusual thought content, hallucinations, disruptions of thought process and self-experience, and the rates of distress and/or impact associated with these experiences. A diverse adolescent and young adult general population sample will be essential. Building off of world-wide efforts with both self-report and interview questions of children and adolescents, items must be written at a pre-adolescent reading level (e.g., fifth grade for U.S. studies) but with as much specificity as possible.
  - Cognitive interviewing, particularly of developmentally and culturally diverse adolescents, is recommended to assist with item wording and to identify the need for developmentally- and culturally-sensitive norms or screen versions (48).
  - Longitudinal data and validation with specialized in-person assessment are needed to identify key self-report questions or sets of questions that might best identify youth at high risk for developing serious mental illness (psychotic and non-psychotic) in the early stages of symptom emergence.
  - Thresholds will need to be defined indicating the appropriateness of a general mental health *versus* a psychosis-specific assessment. Individual risk calculators (31, 49) and resource availability may inform decisions regarding the appropriate level of treatment.
2. Pediatric GP and mental health organizations give rigorous consideration to the development and implementation of broad mental health screens that include probes of psychosis risk, and of guidelines for screening for and responding to psychotic symptoms. Psychosis-specific screening items should be selected based on careful analyses of age, gender, and sociodemographic norms and so as to maximize both sensitivity and specificity of detection (based on progress with step #1 above).
3. Mandated inclusion of material covering the developmental course of major mental illnesses (including risk factors and indicators, screening tools, and clinical management guidelines) in pediatric, family practice, and adult GP and mental health clinician training programs.
4. Large population studies of psychosis screening strategies within pediatric and young adult GP settings to identify best practices and to remove barriers to effective referral and timely assessment and treatment of positive screens. Refinement of clinical staging or stratified care models (50, 51) and expansion of general mental health and specialized care teams are both needed for broad feasibility and to avoid confounding positive GP screens with CHR status.

It is vital to emphasize that help-seeking behavior is not always the primary means of accessing psychosis-specific resources in this population. Research with first-time inpatients with psychosis suggests that roughly half of initial help-seeking is initiated by people other than the ill individual (5). General population screening is intended to enhance early detection of non-help-seeking youth, but it will be important for screening protocols to consider the inclusion of psychosis specific items in screening tools completed by caregivers, teachers, and others in a position to observe early risk indicators.

## Are We Ready to Screen for Psychosis Among General Practitioners? Final Thoughts and Recommendations

The international progress made in identifying individuals at CHR for psychosis and in early intervention in psychosis more broadly, has paved way for a transformation in the roles GP, particularly pediatric and young adult GP, can play in the global healthcare community. They have long been responsible for monitoring and intervening in the health trajectories of young people. Well-child visits provide an opportunity for disclosure and observation that is familiar and which may not carry the same stigma as mental healthcare visits. Policies for mental health screening and treatment may work best if they leverage GP visits to screen for psychosis. Unfortunately, we are not yet ready to screen for psychosis at the population level, particularly in the age range of peak symptom onset. A valid screening tool is needed as the foundation of such an effort, with screening thresholds linked to guidelines on assessment, referral, and intervention. This screening tool must facilitate a stratified approach to screening and subsequent care to maximize the benefit-risk ratio. Such a system would need to provide clear guidelines on graduated assessment and on who to treat and how, providing general care to those who have mild or non-specific risk factors and specialized psychosis resources only to those with specific psychosis risk indicators or established illness.

With both a screening tool and a clear policy, GP can be well positioned to apply their knowledge of patient trajectories to make appropriate referrals, improve rapid response to imminent risk and acute psychosis and support healthy development. In particular, GP have the potential to detect those who are not seeking help through mental health settings. Given their professional orientation toward prevention and early intervention, diagnostic accuracy, and capacity for recognizing syndromes, they are ideal partners in this public health effort.

From a public health perspective, screening has the potential to enhance detection and treatment of psychosis prior to the start of chronic illness. Long-term cost/benefit analysis for well-designed GP psychosis screening programs, including a stratified mental health response, will be an important area for future research. The international work cited paves the way by demonstrating the feasibility and potential effectiveness of GP in this effort. Such innovation is essential to opening up new opportunities for the overall reduction of morbidity and potential prevention of major mental illness.

## Author Contributions

LK conducted the majority of literature review and took the lead in the manuscript organization and writing. KJ provided critical input, research and editing in relation to the public health perspective. JC assisted with the literature review and final manuscript preparation. KW conceived of the manuscript concept, oversaw the literature review and organization of the manuscript, and made a substantial contribution to the writing of the final draft.

## Funding

The open access publication fees came from faculty funds to KW from the Maine Medical Center Research Institute. KW was supported by funding from NIMH (K23MH102358, R21MH116240). KJ was also supported by a grant to Beth Israel Deaconess Medical Center from the Sidney R. Baer, Jr. Foundation.

## Conflict of Interest

The authors declare that the research was conducted in the absence of any commercial or financial relationships that could be construed as a potential conflict of interest.
